# Mitochondrial dysfunction and onset of type 2 diabetes along with its complications: a multi-omics Mendelian randomization and colocalization study

**DOI:** 10.3389/fendo.2024.1401531

**Published:** 2024-08-30

**Authors:** Yang Li, Yahu Miao, Qing Feng, Weixi Zhu, Yijing Chen, Qingqing Kang, Zhen Wang, Fangting Lu, Qiu Zhang

**Affiliations:** Department of Endocrinology, First Affiliated Hospital of Anhui Medical University, Hefei, China

**Keywords:** mitochondrial dysfunction, type 2 diabetes, diabetic complications, Mendelian randomization, colocalization analysis, mitophagy

## Abstract

**Background:**

Mitochondrial dysfunction plays a crucial role in Type 2 Diabetes Mellitus (T2DM) and its complications. However, the genetic pathophysiology remains under investigation. Through multi-omics Mendelian Randomization (MR) and colocalization analyses, we identified mitochondrial-related genes causally linked with T2DM and its complications.

**Methods:**

Summary-level quantitative trait loci data at methylation, RNA, and protein levels were retrieved from European cohort studies. GWAS summary statistics for T2DM and its complications were collected from the DIAGRAM and FinnGen consortiums, respectively. Summary-data-based MR was utilized to estimate the causal effects. The heterogeneity in dependent instrument test assessed horizontal pleiotropy, while colocalization analysis determined whether genes and diseases share the same causal variant. Enrichment analysis, drug target analysis, and phenome-wide MR were conducted to further explore the biological functions, potential drugs, and causal associations with other diseases.

**Results:**

Integrating evidence from multi-omics, we identified 18 causal mitochondrial-related genes. Enrichment analysis revealed they were not only related to nutrient metabolisms but also to the processes like mitophagy, autophagy, and apoptosis. Among these genes, Tu translation elongation factor mitochondrial (*TUFM*), 3-hydroxyisobutyryl-CoA hydrolase (*HIBCH*), and iron-sulfur cluster assembly 2 (*ISCA2*) were identified as Tier 1 genes, showing causal links with T2DM and strong colocalization evidence. *TUFM* and *ISCA2* were causally associated with an increased risk of T2DM, while *HIBCH* showed an inverse causal relationship. The causal associations and colocalization effects for *TUFM* and *HIBCH* were validated in specific tissues. *TUFM* was also found to be a risk factor for microvascular complications in T2DM patients including retinopathy, nephropathy, and neuropathy. Furthermore, drug target analysis and phenome-wide MR underscored their significance as potential therapeutic targets.

**Conclusions:**

This study identified 18 mitochondrial-related genes causally associated with T2DM at multi-omics levels, enhancing the understanding of mitochondrial dysfunction in T2DM and its complications. *TUFM*, *HIBCH*, and *ISCA2* emerge as potential therapeutic targets for T2DM and its complications.

## 1 Introduction

Diabetes Mellitus (DM) has been recognized as a major health concern globally, with its prevalence continually rising, especially in low- and middle-income countries. Type 2 Diabetes Mellitus (T2DM) represents the predominant form of DM, accounting for 90-95% of all diabetes cases (1). Complications of T2DM, including macrovascular and microvascular diseases, diabetic foot, among others, can significantly impair an individual’s quality of life and lifespan (2, 3). The pathogenesis of T2DM primarily involves insulin resistance and dysfunction of pancreatic β-cells. Biological mechanisms leading to cellular insulin resistance include disruptions in insulin signaling pathways, such as the blockage of phosphorylation of insulin receptors and insulin receptor substrates, along with dysfunction in the PI3K and AKT pathways (4). Furthermore, inflammatory cytokines and oxidative stress also contribute to the development of insulin resistance (5). Dysfunctions of pancreatic β-cells are associated with chronic inflammation, oxidative stress, lipotoxicity, and endoplasmic reticulum stress (6, 7). These mechanisms collectively lead to disturbances in human glucose metabolism.

Mitochondria play a pivotal role in regulating energy metabolism, cellular apoptosis, and cell proliferation, thus linking mitochondrial dysfunction with the onset and progression of various diseases. These diseases include metabolic disorders, cardiovascular diseases, neurodegenerative diseases, and certain cancers (8–10). The mechanisms of mitochondrial dysfunction may involve genetic mutations, increased oxidative stress, disturbances in energy metabolism, and aberrant activation of cell death pathways. Mitochondrial dysfunction plays a significant role in T2DM by affecting insulin resistance and pancreatic β-cell function. Firstly, mitochondrial dysfunction disrupts intracellular glucose metabolism, leading to an imbalance in energy metabolism. Secondly, elevated oxidative stress can damage intracellular signaling pathways, impairing the normal action of insulin and causing β-cell dysfunction (11). Lastly, mitochondrial dysfunction affects the oxidation of fatty acids, leading to the accumulation of lipid intermediates, further disrupting insulin signaling pathways and resulting in insulin resistance (12). Additionally, recent studies have identified mitophagy, a specific autophagic process that clears damaged or dysfunctional mitochondria, as influencing insulin resistance and β-cell function (13). However, the biological mechanisms of mitochondrial dysfunction in T2DM require further investigation.

Mendelian Randomization (MR) leverages genetic variations as instrumental variables (IVs) to deduce the causal relationships between exposures and outcomes. This approach adeptly navigates the challenges of reverse causation and confounding factors, which are prevalent in observational studies. The underlying mechanism is the random allocation of genetic variations at the time of conception, effectively mitigating confounders and reverse causality (14). It has been observed that employing cis-variants as IVs may facilitate the balancing of horizontal pleiotropy in the estimation of causal effects between genes and diseases (15). In recent years, thanks to large-scale Genome-Wide Association Study (GWAS) on gene methylation, gene expression, and protein abundance, an increasing number of MR studies have focused on the association between genes and diseases. A recent proteome-wide MR and colocalization analysis identified 11 plasma proteins with colocalization evidence causally linked to T2DM (16). Another bidirectional MR study elucidated the potential mechanisms between T2DM and depression at the gene expression level (17). The causal association between gene methylation and T2DM has also been investigated (18). However, utilizing MR analysis to interpret the potential mechanisms of mitochondrial dysfunction in T2DM and its complications from a multi-omics perspective remains unexplored.

**Figure 1:**
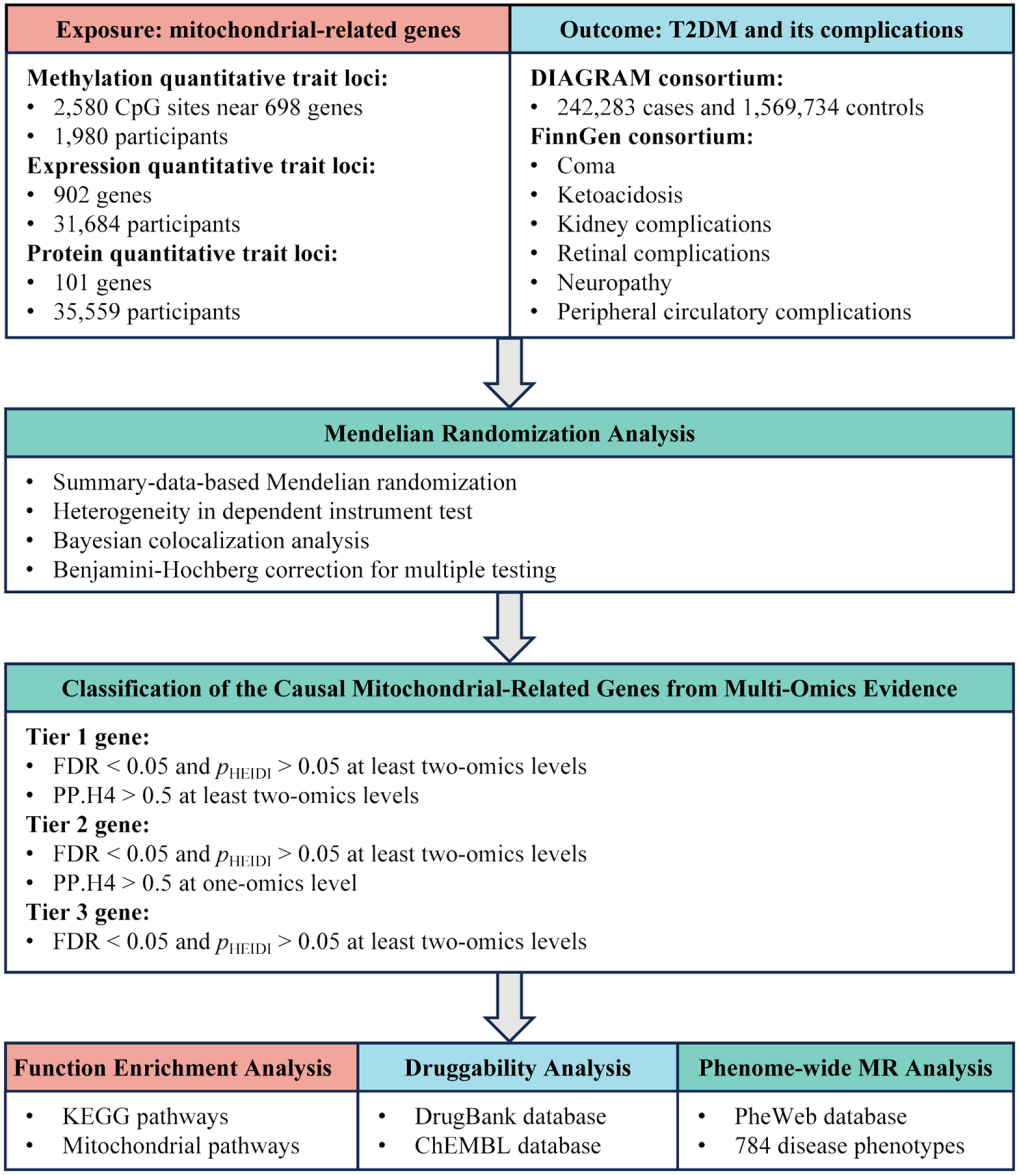
Study design overview for identification of mitochondrial-related genes causally associated with T2DM and its complications by integrating multi-omics evidence.

Utilizing summary-level Quantitative Trait Loci (QTL) data from blood samples at three levels—methylation, RNA, and protein—we employed MR and colocalization analyses to uncover the causal relationships between mitochondrial-related genes and T2DM as well as its complications. The design of this study is displayed in **Figure 1**.

## 2 Materials and methods

### 2.1 Ethical statement

The data in this study is de-identified and collected from the public databases. The approvement and informed consent can be found in the original article cited in this study. Therefore, no further approvement and consent are needed.

### 2.2 Collections of QTL data for gene methylation, gene expression, and protein abundance

Summary-level QTL data could provide IVs representing genetic methylation, expression, and protein levels for MR analysis (**Supplementary Table S1**). Methylation QTL (mQTL) statistics were obtained from the previous study and included blood samples from 1,980 individuals of European ancestry (19). The expression QTL (eQTL) dataset, sourced from the eQTLGen consortium (https://www.eqtlgen.org/), meta-analyzed data of blood samples from 31,684 European individuals (20). Single Nucleotide Polymorphism (SNP)-protein associations were derived from a large-scale protein QTL (pQTL) study based on the deCODE genetics database (https://www.decode.com/summarydata/), encompassing blood samples from 35,559 Icelanders (21). Tissue-specific eQTLs were extracted from the Genotype-Tissue Expression (GTEx) database (https://gtexportal.org/home/) (22). The eQTL datasets from GTEx v8 database were utilized to ascertain the consistency of gene-disease causal relationships and colocalization effects in specific tissues compared to those in blood. In this study, the eQTL datasets for liver, visceral adipose tissue (VAT), pancreas, kidney cortex, tibial nerve, and tibial artery were employed to validate the causal associations and colocalization between gene expression and T2DM, along with its complications.

### 2.3 Collections of summary-level GWAS data for T2DM and its complications

Summary-level GWAS data for T2DM were acquired from the DIAbetes Genetics Replication And Meta-analysis (DIAGRAM) consortium (https://diagram-consortium.org/). This dataset originated from the European ancestry segment of a recent multi-ancestry GWAS on T2DM, comprising 242,283 cases and 1,569,734 controls (23). It encompassed a meta-analysis of extensive European cohorts, including the UK Biobank and FinnGen cohorts, representing the largest European T2DM GWAS dataset to date. In this study, all T2DM diagnoses were standardized using ICD-9 or ICD-10 criteria. For summary-level GWAS data related to T2DM complications, the datasets were collected from the FinnGen R9 database (https://r9.finngen.fi/) (24), including T2DM with coma (ICD-10: E11.0), ketoacidosis (ICD-10: E11.1), kidney complications (ICD-10: E11.2), retinal complications (ICD-10: E11.3), neuropathy (ICD-10: E11.4), and peripheral circulatory complications (ICD-10: E11.5). The number of cases for these datasets range from 657 for ketoacidosis to 4,709 for coma. However, for other T2DM complications like diabetic macrovascular complications, diabetic cardiomyopathy, and diabetic foot, there are currently no suitable public datasets available for MR analysis. Detailed information about these datasets can be found in **Supplementary Table S1**.

### 2.4 Collections of mitochondrial-related genes

MitoCarta3.0 represents a comprehensive mammalian mitochondrial protein database, offering extensive information on mitochondrial proteins (25). This database encompasses 1,136 human protein-coding genes strongly supported for mitochondrial localization. The development of MitoCarta3.0 involved proteomic analysis of mitochondria isolated from 14 different tissues, alongside large-scale assessments of protein localization using green fluorescent protein tagging and microscopic observation. Additionally, the database integrates six other genome-scale mitochondrial localization datasets, employing Bayesian methods to compile this inventory. A notable feature of MitoCarta3.0 is the manual annotation of sub-mitochondrial localization of proteins, including the matrix, inner membrane, intermembrane space, and outer membrane of mitochondria. Furthermore, the database categorizes genes into 149 custom mitochondrial pathways, covering seven major functional categories related to mitochondrial processes. After screening mitochondrial-related genes with available IVs for MR analysis, a total of 698, 902, and 101 genes were identified at the methylation, expression, and protein levels, respectively.

### 2.5 Summary-data-based Mendelian randomization

SMR is a sophisticated analytical method used in genetics and epidemiology to investigate potential causal relationships between traits, typically between QTLs and diseases (26). In this study, we utilized the SMR approach to assess the casual associations of mitochondrial genes methylation, expression, and protein on the susceptibility to T2DM and its complications. We selected top cis-SNP within a 1000 kb range around the gene body or CpG island, achieving the whole genome significance (*p* < 5.0 × 10^-8^). SNPs exhibiting differences in allele frequencies greater than 0.2 between any of the datasets, including the linkage disequilibrium reference, QTL, and outcome data, were excluded to ensure data integrity. To evaluate the IVs pleiotropy, we applied the Heterogeneity in Dependent Instrument (HEIDI) test. A *p*-value of HEIDI test less than 0.05 was indicative of potential pleiotropic effects, leading to the exclusion of the gene from further consideration. These analyses were conducted using the SMR software (version 1.3.1) (26). To control for the false discovery rate (FDR) and maintain its threshold at 0.05, we employed the Benjamini-Hochberg procedure for *p*-value adjustment. Only those associations that passed the FDR-corrected *p*-value threshold and HEIDI test were selected for subsequent colocalization analysis.

### 2.6 Colocalization analysis

In our research, we utilized colocalization analysis to ascertain if the observed causal associations were attributed to linkage disequilibrium. This process was underpinned by a Bayesian framework, which evaluated five distinct scenarios: (a) neither trait is associated; (b) only the first trait is associated; (c) only the second trait is associated; (e) both traits are associated but through separate causal variants; and (f) both traits are associated via a common causal variant (27). Each scenario was assigned a Posterior Probability (PP), denoted as H0 to H4. The analysis parameters included prior probabilities of the SNP’s association with the first trait at 1 × 10^-4^, with the second trait at 1 × 10^-4^, and with both traits at 1 × 10^-5^. A PP.H4 of over 0.7 indicated a strong colocalization, while a PP.H4 between 0.5 and 0.7 suggested a moderate level of colocalization. For the colocalization regions of mQTL-GWAS, we selected a range of 500kb up and down the top cis-SNP (28). For the colocalization regions of eQTL-GWAS and pQTL-GWAS, a range of 1000kb around the gene body was chosen (29). By integrating the results of SMR and colocalization analyses, we can provide more robust evidence for the inference of causal relationships (27). All these analyses were conducted using the ‘coloc’ package (version 5.2.3) in the R software (version 4.1.2) (27).

### 2.7 Classification of the causal mitochondrial-related genes with multi-omics evidence

To comprehensively describe the causal relationships between mitochondrial-related genes and T2DM along with its complications, we integrated results from SMR and colocalization analyses across gene methylation, gene expression, and protein abundance. Based on these results, we categorized causal mitochondrial-related genes into three distinct evidence tiers using the following criteria: (a) ‘Tier 1 genes’ were defined as those exhibiting causal associations with the disease at a minimum of two-omics levels (FDR < 0.05 and *p*_HEIDI_ > 0.05) and concurrently supported by colocalization evidence (PP.H4 > 0.5); (b) ‘Tier 2 genes’ were identified as having causal associations with the disease at least two-omics level (FDR < 0.05 and *p*_HEIDI_ > 0.05) and simultaneously supported by colocalization evidence at one-omics level (PP.H4 > 0.5); (c) ‘Tier 3 genes’ were classified as those showing causal associations with the disease at least two omics levels (FDR < 0.05 and *p*_HEIDI_ > 0.05) but lacking colocalization support (PP.H4 < 0.5). To further investigate the associations among causal mitochondrial-related genes at methylation, RNA, and protein levels, we employed MR and colocalization analyses. These analytical approaches were consistent with those described previously.

### 2.8 Functional enrichment and drug targets analysis

To investigate the potential biological functions of the identified mitochondrial-related genes, we utilized the “clusterProfiler” R package (version 4.8.3) to conduct pathway enrichment analysis (30), including Kyoto Encyclopedia of Genes and Genomes (KEGG) pathways and mitochondrial pathways (25). Following correction for multiple hypotheses testing using the Benjamini-Hochberg method, a more relaxed FDR threshold was adopted (FDR < 0.1) to uncover potentially enriched pathways. The top 10 enriched pathways were selected for visualization. Furthermore, to assess the potential targeted drugs for the causal mitochondrial-related genes, we searched the DrugBank (https://go.drugbank.com/) and ChEMBL (https://www.ebi.ac.uk/chembl/) databases for drugs that target or may target these causal proteins (31, 32).

### 2.9 Phenome-wide MR analysis

To explore the causal relationships of the causal mitochondrial-related genes with other diseases, we conducted a Phe-MR analysis (33). The top cis-SNP of genes at the RNA or protein level was selected as the IV. The Wald ratio method was used to evaluate the causal effects between genes and phenotypes. SNP-disease effect was sourced from the PheWeb database (https://www.leelabsg.org/resources) (34), which utilizes the Scalable and Accurate Implementation of GEneralized mixed model (SAIGE) approach to analyze GWAS data from the UK Biobank cohort, identifying 1403 disease phenotypes based on ICD-9/10 codes. Due to considerations of statistical power, disease phenotypes with fewer than 500 cases were excluded from this study (35). Finally, a total of 784 diseases were included in the Phe-MR analysis. The gene-disease causal effects are considered statistically significant when the FDR is lower than 0.1.

## 3 Results

### 3.1 Causal association between mitochondrial-related gene methylation and T2DM along with its complications

**Figure 2:**
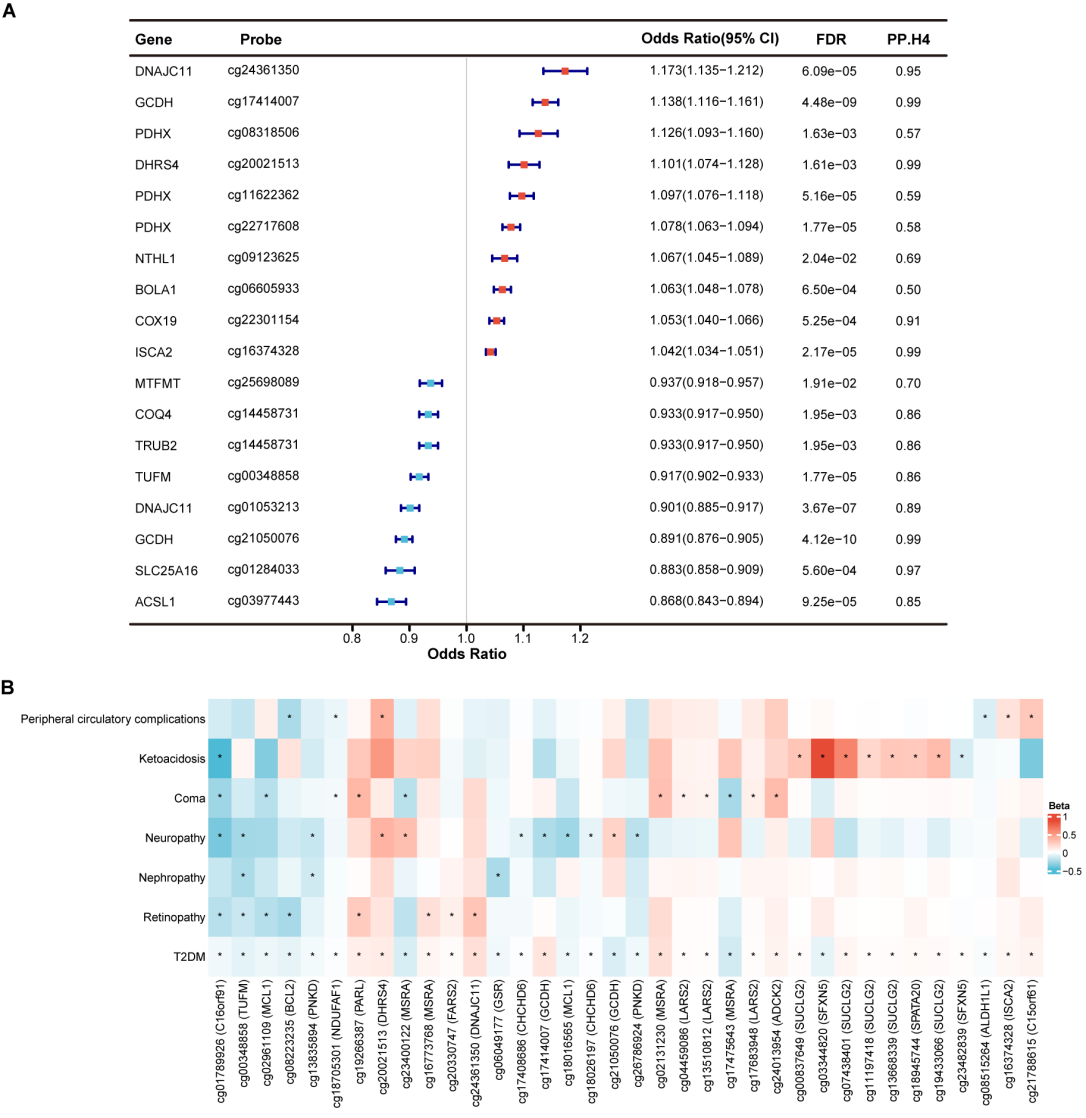
Identification of mitochondrial-related genes causally associated with T2DM and its complications at gene methylation level. **(A)** Forest plot showed the causal mitochondrial-related CpG sites with the support of colocalization evidence. **(B)** Heatmap displayed the causal effects between diabetes-associated mitochondrial CpG sites and T2DM complications. * represents a p-value less than 0.05.

Utilizing a large-scale mQTL dataset derived from blood samples, we employed MR analysis to elucidate the causal associations between methylation of mitochondrial-related genes and T2DM. A total of 2,580 CpG sites near 698 genes were included in the MR analysis. Following multiple hypothesis testing (FDR < 0.05) and exclusion of horizontal pleiotropy (*p*_HEIDI_ > 0.05), 139 CpG sites near 74 genes were identified as having a causal association with T2DM (**Supplementary Table S2**). Among these, 18 CpG sites near 14 genes were supported by colocalization evidence (PP.H4 > 0.5) (**Figure 2A**). Specifically, 13 CpG sites exhibited strong colocalization with T2DM, including *ACSL1* (cg03977443), *COQ4* (cg14458731), *COX19* (cg22301154), *DHRS4* (cg20021513), *DNAJC11* (cg01053213, cg24361350), *GCDH* (cg17414007, cg21050076), *ISCA2* (cg16374328), *MTFMT* (cg25698089), *SLC25A16* (cg01284033), *TRUB2* (cg14458731), and *TUFM* (cg00348858). Other five CpG sites exhibited moderate colocalization, including *BOLA1* (cg06605933), *NTHL1* (cg09123625), and *PDHX* (cg08318506, cg11622362, cg22717608). Genetically predicted each standard deviation (SD) increase in gene methylation, the odds ratios (ORs) for causal effects ranged from 0.868 (95% CI = 0.843-0.894) for cg03977443 (*ACSL1*) to 1.173 (95% CI = 1.135-1.212) for cg24361350 (*DNAJC11*). Moreover, we observed that the causal estimations between different CpG sites within the same gene and T2DM could be directionally inconsistent. For example, within the *GCDH*, methylation at the cg17414007 was associated with an increased risk of T2DM (OR = 1.138, 95% CI = 1.116-1.161), while methylation at the cg21050076 was associated with a decreased risk of T2DM (OR = 0.891, 95% CI = 0.876-0.905).

In exploring the causal relationships between methylation of mitochondrial-related genes and complications of T2DM, we identified numerous CpG sites that surpassed marginal significance (*p* < 0.05) without evidence of horizontal pleiotropy (*p*_HEIDI_ > 0.05). However, only few CpG sites remained significant after FDR correction. Therefore, we focused on the potential causal effects of the identified 139 diabetes-associated CpG sites in T2DM complications, despite most being insignificant after FDR correction. Among these, a total of 36 CpG sites near 22 genes were causally related to at least one of T2DM complication (*p* < 0.05) (**Figure 2B**; **Supplementary Table S3**). CpG site cg01789926 (*C16orf91*) was identified as causally linked to a reduced risk of four T2DM complications, including retinopathy, neuropathy, coma, and ketoacidosis. Methylation at cg00348858 (*TUFM*) was primarily associated with a decreased risk of microvascular complications of T2DM, including retinopathy, nephropathy, and neuropathy. Additionally, cg02961109 (*MCL1*), cg08223235 (*BCL2*), cg13835894 (*PNKD*), cg18705301 (*NDUFAF1*), cg19266387 (*PARL*), cg20021513 (*DHRS4*), and cg23400122 (*MSRA*) were found to be related to at least two T2DM complications, which deserved further investigation. Finally, methylation at *LARS2* (cg04459086, cg13510812 and cg17683948) and *SUCLG2* (cg00837649, cg07438401, cg11197418, cg13668339 and cg19433066) was causally associated with an increased risk of T2DM coma and ketoacidosis, respectively, with consistent directions of causal effects across CpG sites within the same gene.

### 3.2 Causal association between mitochondrial-related gene expression and T2DM along with its complications

**Figure 3:**
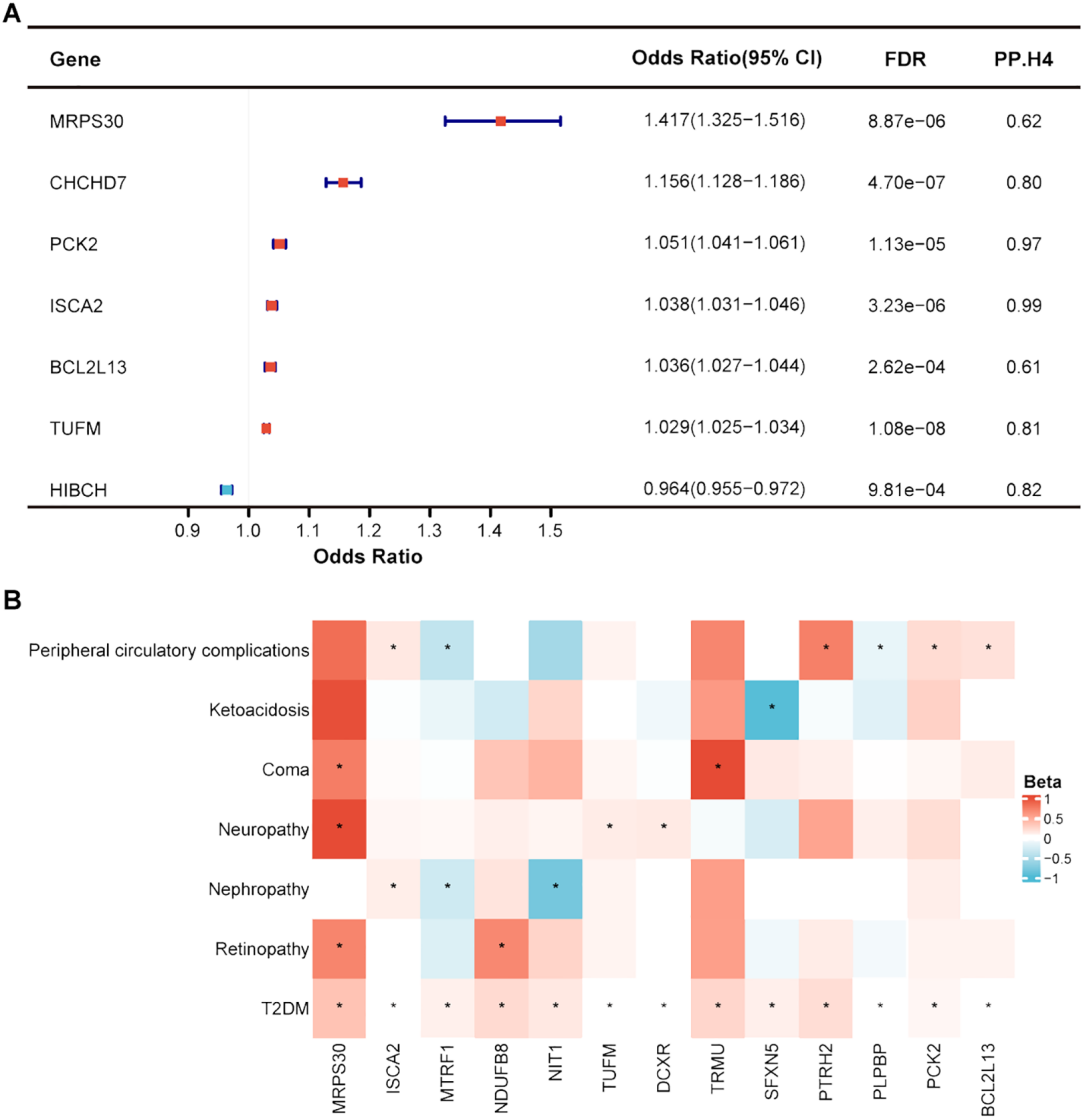
Identification of mitochondrial-related genes causally associated with T2DM and its complications at gene expression level. **(A)** Forest plot showed the causal mitochondrial-related genes with the support of colocalization evidence. **(B)** Heatmap displayed the causal effects between diabetes-associated mitochondrial genes and T2DM complications. * represents a p-value less than 0.05.

To investigate the causal associations between mitochondrial-related genes and T2DM as well as its complications at the RNA level, we utilized eQTL data from blood samples of 31,684 participants for MR analysis. A total of 902 mitochondrial-related genes were included in the MR analysis, 44 of which were identified as causal genes after FDR correction (FDR < 0.05) and exclusion of horizontal pleiotropy (*p*_HEIDI_ > 0.05) (**Supplementary Table S4**). Only seven genes were supported by colocalization evidence (PP.H4 > 0.5), including *ISCA2*, *PCK2*, *HIBCH*, *TUFM*, and *CHCHD7* with high support, and *MRPS30* and *BCL2L13* with moderate support (**Figure 3A**). For each SD increase in gene expression at the RNA level, the ORs for causal effects ranged from 0.792 (95% CI = 0.744-0.843) for *FPGS* to 1.426 (95% CI = 1.284-1.583) for *RDH14*.

When exploring the causal relationships between RNA-level expression of mitochondrial-related genes and T2DM complications, few genes remained significant after FDR correction, despite identifying many that surpassed marginal significance (*p* < 0.05) without evidence of horizontal pleiotropy (*p*_HEIDI_ > 0.05). As previously mentioned, we focused on the potential causal effects of the identified 44 diabetes-associated genes at the RNA level in T2DM complications (**Supplementary Table S5**). Thirteen genes were causally associated with at least one T2DM complication, among which *MRPS30*, *ISCA2*, and *MTRF1* were related to at least two T2DM complications (**Figure 3B**). Increased expression of *MRPS30* was predicted to be a risk factor for retinopathy, neuropathy, and coma in T2DM patients. *ISCA2* expression at RNA level was positively correlated with T2DM nephropathy and peripheral circulatory diseases. However, the causal direction of *MTRF1* with T2DM nephropathy and peripheral circulatory diseases was inconsistent with T2DM, warranting cautious interpretation of these results.

### 3.3 Causal association between mitochondrial-related plasma protein and T2DM along with its complications

**Figure 4:**
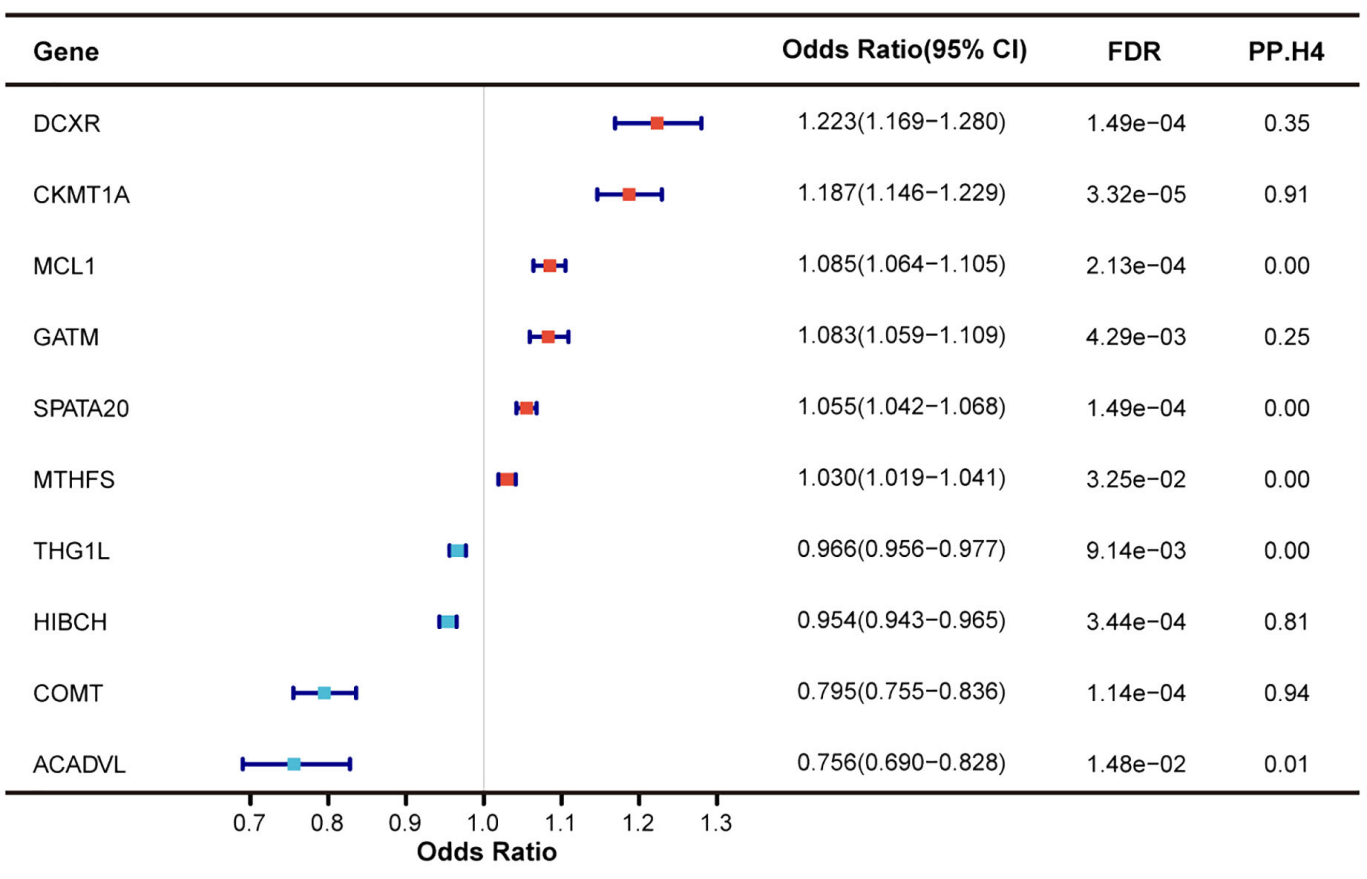
Identification of mitochondrial-related genes causally associated with T2DM at protein level.

Utilizing large-scale plasma pQTL data from the deCODE genetics, we applied SMR and colocalization analyses to reveal the causal association between mitochondrial-related plasma proteins and T2DM. Among 101 mitochondrial-related plasma proteins, only 10 plasma proteins were identified as having a causal association with T2DM (FDR < 0.05) without evidence of horizontal pleiotropy (*p*_HEIDI_ > 0.05) (**Figure 4**). Plasma proteins COMT, CKMT1A, and HIBCH exhibited high support of colocalization with T2DM, deserving further investigation. For each SD increase in genetically predicted plasma protein levels, the ORs for causal effects ranged from 0.756 (95% CI = 0.690-0.828) for ACADVL to 1.223 (95% CI = 1.169-1.280) for DCXR (**Supplementary Table S6**).

Regarding the causal associations between mitochondrial-related genes at the protein level and T2DM complications, only few plasma proteins exceeded marginal significance (*p* < 0.05) without evidence of horizontal pleiotropy (*p*_HEIDI_ > 0.05), though none passed FDR correction (**Supplementary Table S7**). Among the diabetes-associated plasma proteins, only those encoded by *COMT* and *DCXR* showed a potential causal effect with T2DM complications (*p* < 0.05). A decrease in genetically predicted COMT plasma abundance was causally associated with an increased risk of T2DM nephropathy and coma, aligning with the causal direction between COMT and T2DM. However, no colocalization evidence was found between COMT and T2DM nephropathy and coma, despite high support of colocalization between COMT and T2DM. DCXR was found to be causally associated with T2DM nephropathy, yet the causal direction was inconsistent with T2DM, suggesting potential bias.

### 3.4 Identifying causal mitochondrial-related genes from multi-omics evidence

In this study, we integrated the results of MR and colocalization analyses from gene methylation, gene expression, and protein abundance with T2DM in blood, identifying 18 causal mitochondrial-related genes. These included 3 Tier 1 genes, 2 Tier 2 genes, and 13 Tier 3 genes (**Table 1**). *TUFM*, *ISCA2*, and *HIBCH* were classified as Tier 1 genes. Methylation at cg00348858 within the *TUFM* was associated with a decreased risk of T2DM, while methylation at cg16374328 within the *ISCA2* was linked to an increased risk (**Table 1**). *HIBCH* lacked data on gene methylation levels. Increased RNA expression of *TUFM* and *ISCA2* in blood was predicted to be a risk factor for T2DM, whereas an increase in *HIBCH* was protective. Only *HIBCH* had data supporting MR analysis at the protein level, suggesting its protective role in T2DM. High support of colocalization evidence further confirmed the causal associations between Tier 1 genes and T2DM (**Table 1**). Additionally, we employed MR and colocalization analyses to explore the causal relationships across methylation, RNA, and protein levels (**Supplementary Table S8**). Methylation at cg00348858 in the *TUFM* was causally linked to a decrease in RNA expression, fitted with the causal linkage between cg00348858 methylation and its RNA expression with T2DM. Conversely, methylation at cg16374328 in the *ISCA2* positively correlated with its RNA expression, also aligning with previous findings. A positive correlation was found between RNA expression and protein abundance for *HIBCH*. Colocalization evidence was only found in *ISCA2* (PP.H4 = 0.99) and *HIBCH* (PP.H4 = 0.98), making the causality more reliable (**Supplementary Table S8**). Besides, Tier 2 genes *COX19* and *COMT* showed causal associations with T2DM at two-omics levels and colocalization evidence at only one-omics level. Tier 3 genes, including *GATM*, *DCXR*, and *SPATA20*, were associated with T2DM across all three-omics levels without colocalization evidence. The relationships between these genes at methylation, RNA, and protein levels were consistent with their causal effects on T2DM (**Supplementary Table S8**).

**Table 1:** The causal mitochondrial-related gene identified by integrating evidence from multi-omics.

Gene	Tier	mQTL-T2DM				eQTL-T2DM			pQTL-T2DM		
		Probe	OR (95% CI)	FDR	PP.H4	OR (95% CI)	FDR	PP.H4	OR (95% CI)	FDR	PP.H4
TUFM	Tier 1	cg00348858	0.917 (0.902-0.933)	1.77 × 10^-5^	0.86	1.029 (1.025-1.034)	1.08 × 10^-8^	0.81			
ISCA2	Tier 1	cg16374328	1.042 (1.034-1.051)	2.17 × 10^-5^	0.99	1.038 (1.031-1.046)	3.23 × 10^-6^	0.99			
HIBCH	Tier 1					0.964 (0.955-0.972)	9.81 × 10^-4^	0.82	0.954 (0.943-0.965)	3.44 × 10^-4^	0.81
COX19	Tier 2	cg22301154	1.053 (1.040-1.066)	5.25 × 10^-4^	0.91	1.056 (1.036-1.076)	3.00 × 10^-2^	1.35 × 10^-17^			
COMT	Tier 2	cg19930203	1.051 (1.032-1.071)	4.88 × 10^-2^	5.58 × 10^-3^				0.795 (0.755-0.836)	1.14 × 10^-4^	0.94
GATM	Tier 3	cg11431346	0.926 (0.902-0.951)	3.21 × 10^-2^	0.33	1.025 (1.018-1.033)	7.98 × 10^-3^	0.25	1.083 (1.059-1.109)	4.29 × 10^-3^	0.25
DCXR	Tier 3	cg07073120	0.933 (0.913-0.953)	1.71 × 10^-2^	0.37	1.020 (1.014-1.026)	1.02 × 10^-2^	0.01	1.223 (1.169-1.280)	1.49 × 10^-4^	0.35
SPATA20	Tier 3	cg16020904	0.917 (0.895-0.940)	6.22 × 10^-3^	4.14 × 10^-4^	1.017 (1.013-1.021)	2.58 × 10^-4^	6.43 × 10^-4^	1.055 (1.043-1.068)	1.49 × 10^-4^	5.27 × 10^-4^
MRPL32	Tier 3	cg00365680	0.979 (0.972-0.987)	4.25 × 10^-2^	0.11	0.946 (0.927-0.966)	4.72 × 10^-2^	1.17 × 10^-5^			
HADHA	Tier 3	cg08067268	0.981 (0.975-0.988)	2.68 × 10^-2^	8.65 × 10^-6^	1.062 (1.042-1.083)	1.62 × 10^-2^	1.05 × 10^-9^			
AIFM2	Tier 3	cg04859918	0.969 (0.959-0.980)	4.61 × 10^-2^	1.04 × 10^-13^	0.947 (0.930-0.965)	2.35 × 10^-2^	1.74 × 10^-19^			
SFXN5	Tier 3	cg03344820	0.925 (0.901-0.949)	2.62 × 10^-2^	0.01	1.097 (1.069-1.125)	3.80 × 10^-3^	0.01			
STYXL1	Tier 3	cg03592824	1.030 (1.020-1.039)	1.90 × 10^-2^	0.22	1.015 (1.010-1.020)	1.96 × 10^-2^	1.74 × 10^-6^			
ACSM3	Tier 3	cg06478823	1.019 (1.013-1.026)	2.83 × 10^-2^	9.39 × 10^-6^	0.930 (0.908-0.953)	2.35 × 10^-2^	7.73 × 10^-6^			
SLC25A13	Tier 3	cg21022364	0.973 (0.965-0.981)	1.12 × 10^-2^	0.32	1.042 (1.029-1.055)	9.24 × 10^-3^	0.24			
MTHFS	Tier 3					1.033 (1.022-1.045)	3.43 × 10^-2^	2.69 × 10^-3^	1.030 (1.019-1.041)	3.25 × 10^-2^	2.30 × 10^-3^
MCL1	Tier 3	cg02961109	0.946 (0.929-0.964)	2.87 × 10^-2^	5.75 × 10^-6^				1.085 (1.064-1.105)	2.13 × 10^-4^	5.57 × 10^-9^
ACADVL	Tier 3	cg03508063	1.066 (1.046-1.087)	1.16 × 10^-2^	3.54 × 10^-3^				0.756 (0.690-0.828)	1.48 × 10^-2^	0.01

### 3.5 Tissue-specific validation of the causal mitochondrial-related genes at the gene expression level

For T2DM, we validated the causal associations and colocalization effects between T2DM and 18 causal mitochondrial-related genes identified at the multi-omics level using blood samples, particularly in specific tissues such as liver, pancreas, and VAT (**Supplementary Table S9**). Of these, only six genes were validated in specific tissues, including *HIBCH*, *SPATA20*, *STYXL1*, *TUFM*, *MTHFS*, and *DCXR* (**Table 2**). Initially, increased expression of *SPATA20* and *STYXL1* in the three tissues was causally linked to an elevated risk of T2DM, with an opposite causal effect observed for *HIBCH*. Only *HIBCH* was supported by colocalization evidence in the liver (PP.H4 = 0.85), pancreas (PP.H4 = 0.76), and VAT (PP.H4 = 0.76). Subsequently, *TUFM* exhibited a positive causal relationship with T2DM in the pancreas (PP.H4 = 0.82) and VAT (PP.H4 = 0.85), with colocalization effects. However, the causal effects of *MTHFS* on T2DM varied between the pancreas (OR = 0.972, 95% CI = 0.958-0.986) and VAT (OR = 1.017, 95% CI = 1.009-1.025), warranting further investigation. Additionally, *DCXR* expression was only found to have a causal relationship with T2DM in VAT. Finally, the other causal mitochondrial-related genes lacked tissue-specific validation.

**Table 2:** Validation of the causal mitochondrial-related genes in specific tissues at the gene expression level.

Gene	Tissue	OR (95% CI)	P-value	P-HEIDI	PP.H4
HIBCH	Liver	0.975 (0.969-0.981)	7.99 × 10^-5^	0.467	0.85
HIBCH	Pancreas	0.966 (0.957-0.975)	1.71 × 10^-4^	0.058	0.76
HIBCH	Visceral adipose tissue	0.954 (0.943-0.966)	1.40 × 10^-4^	0.318	0.76
SPATA20	Liver	1.018 (1.013-1.024)	6.65 × 10^-4^	0.007	2.74 × 10^-4^
SPATA20	Pancreas	1.027 (1.020-1.033)	2.15 × 10^-5^	0.058	5.81 × 10^-4^
SPATA20	Visceral adipose tissue	1.019 (1.015-1.024)	1.25 × 10^-5^	0.068	5.81 × 10^-4^
STYXL1	Liver	1.028 (1.017-1.039)	8.85 × 10^-3^	0.432	0.01
STYXL1	Pancreas	1.026 (1.017-1.035)	5.11 × 10^-3^	0.203	1.66 × 10^-6^
STYXL1	Visceral adipose tissue	1.020 (1.013-1.026)	2.11 × 10^-3^	0.098	1.72 × 10^-6^
TUFM	Pancreas	1.056 (1.045-1.066)	2.76 × 10^-8^	0.024	0.82
TUFM	Visceral adipose tissue	1.228 (1.18-1.279)	3.60 × 10^-7^	0.131	0.85
MTHFS	Pancreas	0.972 (0.958-0.986)	4.23 × 10^-2^	0.173	1.75 × 10^-3^
MTHFS	Visceral adipose tissue	1.017 (1.009-1.025)	2.76 × 10^-2^	0.163	7.40 × 10^-4^
DCXR	Visceral adipose tissue	1.112 (1.071-1.155)	4.73 × 10^-3^	0.284	7.74 × 10^-3^

Regarding T2DM complications, the potential mitochondrial-related genes previously identified (**Supplementary Tables S3**, **S5**, **S7**) were subjected to tissue-specific validation for causal associations (**Supplementary Table S9**). In the tibial nerve, increased expression of *DCXR*, *GCDH*, and *TUFM* was identified as risk factors for T2DM neuropathy. In the tibial artery, genetically predicted increased expression of *GSTZ1* and *PCK2* was causally linked to an increased risk of peripheral circulatory complications in T2DM patients, while *HAGH* exhibited an opposite causal effect. *HEBP1* expression in the liver was positively related to ketoacidosis in T2DM patients. However, evidence supporting tissue-specific validation for T2DM retinopathy, nephropathy, and coma is still lacking.

### 3.6 Functional enrichment analysis of the identified mitochondrial-related genes in T2DM

**Figure 5:**
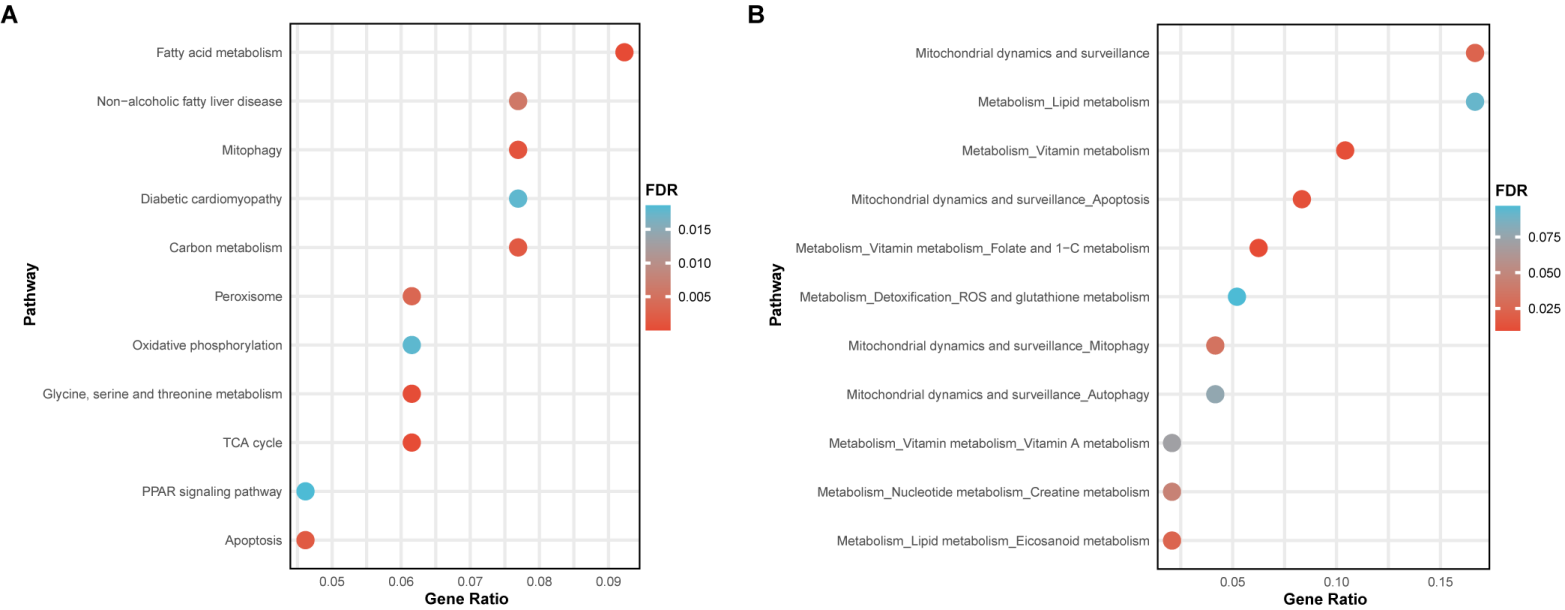
Pathway enrichment analysis of the identified mitochondrial-related genes. **(A)** Bubble plot showed the enrichment results of KEGG pathways. **(B)** Bubble plot displayed the enrichment results of mitochondrial pathways.

For pathway enrichment analysis, mitochondrial-related genes showing a causal relationship (FDR < 0.05 and *p*_HEIDI_ > 0.05) at any level—methylation (**Supplementary Table S2**), RNA (**Supplementary Table S4**), or protein (**Supplementary Table S6**) in T2DM—were included, with a special focus on KEGG pathways and mitochondrial pathways. The enrichment analysis of KEGG pathways indicated that these identified mitochondrial-related genes were associated not only with various nutrient metabolisms, such as carbohydrates, fatty acids, and amino acids, but also with key cellular biological functions like mitophagy, apoptosis, and the PPAR signaling pathway. Moreover, two diseases highly related to T2DM, non-alcoholic fatty liver disease and diabetic cardiomyopathy, were also enriched (**Figure 5A**). Regarding mitochondrial pathways, it was found that the identified mitochondrial-related genes predominantly enriched in mitochondrial metabolism and mitochondrial dynamics and surveillance. Within mitochondrial metabolism, these genes may influence lipid, vitamin, nucleotide metabolism, and detoxification. Concurrently, they might also affect mitochondrial dynamics and surveillance, especially mitophagy, autophagy, and apoptosis, aligning with the enriched KEGG pathways observed previously (**Figure 5B**).

### 3.7 Druggability of the causal mitochondrial-related genes

Through searching the DrugBank and ChEMBL databases, we explored the potential drugs of the 18 causal mitochondrial-related genes previously identified. Among these genes, only 8 were linked to relevant targeted drugs, including *TUFM*, *HIBCH*, *COMT*, *GATM*, *DCXR*, *HADHA*, *SLC25A13*, and *MTHFS* (**Supplementary Table S10**). It was discovered that zinc acts as a cofactor for TUFM, thereby influencing its biological functions. Quercetin has been identified as targeting HIBCH, though its effects remain unclear. A variety of inhibitors, substrates, and inducers are known to affect the activity of COMT. As a protective factor for T2DM, the inducer Nylidrin for COMT may represent a potential therapeutic option for T2DM. The specific actions for the targeted drugs related to the remaining five druggable genes are still under investigation.

### 3.8 Phe-MR analysis of the causal mitochondrial-related genes

To assess the beneficial or detrimental effects of the causal mitochondrial-related genes on other diseases, we employed the Phe-MR analysis to investigate the causal relationships between these genes and 784 disease phenotypes collected from the UK Biobank. A total of 849 gene-disease associations reached marginal significance (*p* < 0.05) (**Supplementary Table S11**). However, only 13 causal associations remained statistically significant after FDR correction (FDR < 0.1) (**Table 3**). *TUFM*, a risk factor for T2DM, was positively associated not only with obesity and overweight but also with varicose veins and rheumatoid arthritis, underscoring the importance as a therapeutic target. *ACADVL* was found to be associated with a reduced risk of essential hypertension, nonspecific chest pain, and skin non-epithelial cancer. Therefore, targeting *ACADVL* may be beneficial for patients with T2DM who also suffer from hypertension. Targeting *SLC25A13*, while potentially lowering the risk of T2DM, may lead to neutropenia and a range of musculoskeletal diseases, such as arthropathy, intervertebral disc degeneration, and joints disorders.

**Table 3:** The Phe-MR results of the causal mitochondrial-related genes after FDR correction.

Gene	Disease	SNP	Effect allele	Other allele	OR (95% CI)	FDR
TUFM	Obesity	rs7187776	G	A	1.084 (1.049-1.119)	6.33 × 10^-4^
TUFM	Overweight	rs7187776	G	A	1.081 (1.047-1.117)	6.33 × 10^-4^
TUFM	Varicose veins	rs7187776	G	A	1.078 (1.044-1.114)	9.48 × 10^-4^
TUFM	Rheumatoid arthritis	rs7187776	G	A	1.089 (1.037-1.143)	9.50 × 10^-2^
MCL1	Nasal polyps	rs41272019	T	C	0.586 (0.469-0.732)	2.04 × 10^-3^
ACADVL	Non-epithelial cancer of skin	rs507506	G	A	0.326 (0.185-0.576)	2.99 × 10^-2^
ACADVL	Essential hypertension	rs507506	G	A	0.494 (0.379-0.643)	6.61 × 10^-5^
ACADVL	Nonspecific chest pain	rs507506	G	A	0.526 (0.369-0.749)	7.17 × 10^-2^
SLC25A13	Disorders of the pituitary gland and its hypothalamic control	rs17167473	A	G	1.850 (1.304-2.625)	7.36 × 10^-2^
SLC25A13	Neutropenia	rs17167473	A	G	0.747 (0.634-0.879)	7.36 × 10^-2^
SLC25A13	Arthropathy	rs17167473	A	G	0.912 (0.865-0.961)	7.36 × 10^-2^
SLC25A13	Degeneration of intervertebral disc	rs17167473	A	G	0.713 (0.598-0.849)	5.82 × 10^-2^
SLC25A13	Symptoms and disorders of the joints	rs17167473	A	G	0.740 (0.636-0.860)	5.82 × 10^-2^

## 4 Discussion

In this study, we employed MR and colocalization analysis to uncover the causal associations between mitochondrial-related genes in blood at the levels of methylation, RNA, and protein with T2DM and its complications. By integrating multi-omics evidence of MR and colocalization results, 18 causal mitochondrial-related genes were identified and validated in specific tissues. We also investigated the underlying regulation network of these genes at multi-omics levels. Furthermore, we delved deeper into the potential functions, their druggability, and associations with other diseases through enrichment analysis, targeted drug searching, and Phe-MR analysis, respectively.

*TUFM* encodes a protein known as mitochondrial Tu translation elongation factor, which plays a pivotal role in the mitochondrial protein biosynthesis by facilitating the binding of aminoacyl-tRNA to ribosomes. Mitochondria produce ATP through oxidative phosphorylation, a process contingent upon the synthesis of proteins within the mitochondria. Therefore, *TUFM* and its encoded protein are essential for maintaining cellular energy metabolism and functionality. In recent years, *TUFM* has been identified as playing significant roles in mitophagy (36) and autophagy (37) and is associated with diseases such as non-alcoholic fatty liver disease (38), Alzheimer’s disease (39), and multiple sclerosis (40). However, the relationship between *TUFM* and T2DM, along with its complications, remains unclear. Utilizing MR and colocalization analysis, *TUFM* was revealed to have a causal relationship with increased risk of T2DM at the methylation and RNA levels, supported by colocalization evidence. This causal effect and colocalization evidence have also been validated in the pancreas and VAT. Furthermore, *TUFM* has been found to potentially have a causal association with microvascular complications of T2DM, including retinopathy, nephropathy, and neuropathy, warranting further investigation. Additionally, druggability analysis suggested that zinc can act as a cofactor, influencing the biological function of TUFM. Phe-MR revealed that *TUFM* was also a risk factor for obesity, overweight, varicose veins, and rheumatoid arthritis, emphasizing its significance as a drug target candidate.

The protein encoded by *HIBCH* functions as 3-hydroxyisobutyryl-CoA hydrolase, a mitochondrial enzyme involved in valine metabolism. Deficiencies in *HIBCH* have been linked to a series of neurological disorders, including developmental delays, movement disorders, abnormal muscle tone, and cognitive impairments (41). Recent studies have highlighted its significant roles in colorectal cancer and fatty liver disease. Elevated *HIBCH* expression negatively correlates with the survival of colorectal cancer patients and is associated with tumor proliferation, anti-apoptosis, and reduced autophagy (42). In hepatocytes, *HIBCH* expression positively correlates with liver fat content, inhibits oxidative phosphorylation, and enhances reactive oxygen species (ROS) production, mechanisms that potentially linked to metabolic diseases (43). In our study, genetically predicted increased expression of *HIBCH* at RNA and protein levels was causally associated with a reduced risk of T2DM, supported by colocalization evidence. This causal association and colocalization effect were also validated in the liver, pancreas, and VAT. However, there is no evidence to support a causal relationship between *HIBCH* and T2DM complications. The potential associations between *HIBCH* and other diseases remain unclear, and targeted drugs are still under development.

*ISCA2* encodes the iron-sulfur cluster assembly 2 protein, playing a crucial role in the biosynthesis of cellular iron-sulfur (Fe-S) clusters. Fe-S clusters are complexes ubiquitously present in organisms, essential for numerous cellular processes, including electron transport in the respiratory chain, regulation of enzyme activity, nucleic acid metabolism, and protein synthesis. Deficiencies in *ISCA2* manifest as a rare hereditary disease characterized by infantile-onset leukodystrophy and neurodegenerative changes, leading to early childhood mortality in most cases (44). Moreover, *ISCA2* inhibition in clear cell renal cell carcinoma has been shown to decrease HIF-1/2α levels and induce ferroptosis by disrupting iron metabolism (45). The loss of function of *ISCA2* disrupts the formation of (Fe-S) clusters, impairing mitochondrial function, increasing ROS, and hindering erythroid differentiation and cell proliferation (46). However, no studies have yet indicated a correlation between *ISCA2* and T2DM as well as its complications. Integrating methylation and RNA-level MR and colocalization results, *ISCA2* has been revealed to have a positive causal association with T2DM, which was not validated in the liver, pancreas, and VAT. Additionally, at the RNA level, elevated expression of *ISCA2* potentially increased the risk of nephropathy and peripheral circulatory complications in T2DM patients.

Beyond *TUFM*, *HIBCH*, and *ISCA2*, Tier 2 and Tier 3 genes also play significant roles in T2DM. In high-fat diet mouse models, using COMT inhibitors exacerbated glucose intolerance, led to increased lipid deposition in the liver, and enhanced macrophage accumulation (47). AIFM2 functions as an enzyme oxidizing NADH to sustain elevated cytosolic NAD levels, facilitating vigorous glycolysis and electron transfer to the electron transport chain, thus combating diet-induced obesity and insulin resistance (48). ACSM3, a mitochondrial lipid metabolism enzyme in the liver, is significantly reduced in patients with metabolic syndrome, and systemic or liver-specific depletion of *Acsm3* in mice results in metabolic syndrome (49). The roles of these genes align with the direction of causal associations observed in our study, further affirming the reliability of our research. However, other causal mitochondrial-related genes are still under investigation. Despite the lack of experimental evidence, our findings still offer suggestive insights. We identified that *COX19*, *GATM*, *DCXR*, *SPATA20*, *HADHA*, *SFXN5*, *STYXL1*, *SLC25A13*, *MTHFS*, and *MCL1* have a causal association with an increased risk of T2DM, while inverse causal effects were found in *MRPL32* and *ACADVL*.

Utilizing enrichment analysis of KEGG pathways and mitochondrial pathways, the identified mitochondrial-related genes and their potential mechanisms in T2DM were explored. These genes were implicated not only in various nutrient metabolisms, such as carbohydrates, fatty acids, and amino acids, but also in mitochondrial dynamics and surveillance, including mitophagy, autophagy, and apoptosis. Additionally, two diseases closely associated with T2DM, non-alcoholic fatty liver disease and diabetic cardiomyopathy, were also enriched. Given the critical roles of pancreatic β-cell dysfunction and insulin resistance in T2DM, we hypothesize that these genes may contribute to β-cell dysfunction and insulin resistance not only by directly affecting nutrient metabolisms but also through mitophagy, autophagy, and apoptosis (11–13). This further emphasizes the significance of studying mitochondrial dysfunction, particularly mitophagy, autophagy, and apoptosis, in T2DM and its complications.

The strength of this study lies in our combination of MR and colocalization analyses to explore the causal relationships between mitochondrial-related genes and T2DM along with its complications. MR analysis minimizes biases from confounding factors and reverse causality, while colocalization analysis addresses potential biases due to linkage disequilibrium. Additionally, we not only integrated results from methylation, RNA, and protein levels but also validated these associations in specific tissues, further reinforcing the evidence for causality. However, recognizing the limitations aids in correctly interpreting the results. Firstly, the analysis was limited to European ancestry, reducing the generalizability of our findings to other populations. Secondly, the limited sample sizes in GWAS cohorts at the levels of methylation, RNA, and protein may introduce potential biases into the results. Thirdly, we utilized the largest GWAS cohort of T2DM in European populations to date. Since it encompasses the majority of European T2DM cohorts in a meta-analysis, there was no suitable dataset for validation. Fourth, the datasets related to T2DM complications were still limited in terms of diseases and sample sizes, suggesting caution in interpreting the causal relationships. Fifth, to mitigate horizontal pleiotropy, cis-SNPs were used as IVs, possibly overlooking the effects of trans-regulation, which might be crucial for some protein-coding genes. Sixth, this study may have ignored some meaningful genes lacking suitable IVs for MR analysis. Seventh, our study primarily utilized QTL datasets derived from nuclear DNA, excluding mitochondrial DNA, which could potentially omit important genetic contributors to mitochondrial dysfunction in T2DM. Last but not least, these causal associations are based solely on MR and colocalization analyses, thus requiring further validation through population-based studies and *in vitro*/*in vivo* experiments.

In summary, this study integrated MR and colocalization analyses to identify 18 causal mitochondrial-related genes associated with T2DM across methylation, RNA, and protein levels, and validated them in specific tissues. Among these genes, *TUFM*, *HIBCH*, and *ISCA2* could serve as biomarkers and potential therapeutic targets for mitochondrial therapy in T2DM. These findings highlight the crucial role of mitochondrial dysfunction in the pathogenesis of T2DM and its complications.

## Data Availability

Publicly available datasets were analyzed in this study. The datasets used and provided here are available for download from various digital archives. Both the repository(s) and accession number(s) are listed in the article/**Supplementary Materials**.

## Glossary

T2DM: Type 2 diabetes mellitus
MR: Mendelian randomization
PI3K: Phosphoinositide 3-kinases
IV: Instrumental variable
GWAS: Genome-wide association study
QTL: Quantitative trait loci
SNP: Single nucleotide polymorphism
GTEx: Genotype-tissue expression
VAT: Visceral adipose tissue
DIAGRAM: Diabetes genetics replication and meta-analysis
ICD: International classification of diseases
SMR: Summary-data-based Mendelian randomization
HEIDI: Heterogeneity in dependent instrument
FDR: False discovery rate
PP: Posterior probability
KEGG: Kyoto encyclopedia of genes and genomes
Phe-MR: Phenome-wide Mendelian randomization
SAIGE: Scalable and accurate implementation of generalized mixed model
ROS: Reactive oxygen species
TUFM: Tu Translation Elongation Factor, Mitochondrial
ISCA2: Iron-Sulfur Cluster Assembly 2
HIBCH: 3-Hydroxyisobutyryl-CoA Hydrolase
COX19: Cytochrome C Oxidase Assembly Factor COX19
COMT: Catechol-O-Methyltransferase
GATM: Glycine Amidinotransferase (L-arginine:glycine amidinotransferase)
DCXR: Dicarbonyl And L-Xylulose Reductase
SPATA20: Spermatogenesis Associated 20
MRPL32: Mitochondrial Ribosomal Protein L32
HADHA: Hydroxyacyl-CoA Dehydrogenase Trifunctional Multienzyme Complex Subunit Alpha
AIFM2: Apoptosis Inducing Factor Mitochondria Associated 2
SFXN5: Sideroflexin 5
STYXL1: Serine/Threonine/Tyrosine Interacting Like 1
ACSM3: Acyl-CoA Synthetase Medium-Chain Family Member 3
SLC25A13: Solute Carrier Family 25 Member 13 (Citrin)
MTHFS: Methenyltetrahydrofolate Synthetase
MCL1: Myeloid Cell Leukemia 1
ACADVL: Acyl-CoA Dehydrogenase Very Long Chain
C16orf91: Chromosome 16 Open Reading Frame 91
BCL2: B-Cell CLL/Lymphoma 2
PNKD: Paroxysmal Non-Kinesigenic Dyskinesia
NDUFAF1: NADH: Ubiquinone Oxidoreductase Complex Assembly Factor 1
PARL: Presenilin Associated Rhomboid Like
DHRS4: Dehydrogenase/Reductase 4
MSRA: Methionine Sulfoxide Reductase A
LARS2: Leucyl-tRNA Synthetase 2, Mitochondrial
SUCLG2: Succinate-CoA Ligase, GDP-Forming, Beta Subunit
MRPS30: Mitochondrial Ribosomal Protein S30
MTRF1: Mitochondrial Translation Release Factor 1
GCDH: Glutaryl-CoA Dehydrogenase
GSTZ1: Glutathione S-Transferase Zeta 1
PCK2: Phosphoenolpyruvate Carboxykinase 2
HAGH: Hydroxyacylglutathione Hydrolase
HEBP1: Heme Binding Protein 1
ATP: Adenosine Triphosphate
HIF: Hypoxia-Inducible Factor
NAD/NADH: Nicotinamide Adenine Dinucleotide.
