# Supplementary material for: Mitochondrial dysfunction and onset of type 2 diabetes along with its complications: a multi-omics Mendelian randomization and colocalization study
Source: Front Endocrinol (Lausanne). 2024 Aug 30;15:1401531. doi: 10.3389/fendo.2024.1401531 (PMC11392782; doi:10.3389/fendo.2024.1401531)
Supplement: Supplementary file 2 [file DataSheet1.docx]

**STROBE-MR checklist of recommended items to address in reports of Mendelian randomization studies**^1^ ^2^

| **Item No.** | **Section** | **Checklist item** |  | **Relevant text from manuscript** |
| --- | --- | --- | --- | --- |
| 1 | **TITLE and ABSTRACT** | Indicate Mendelian randomization (MR) as the study’s design in the title and/or the abstract if that is a main purpose of the study |  | Mitochondrial dysfunction and onset of type 2 diabetes along with its complications: a multi-omics Mendelian randomization and colocalization study |
|  | **INTRODUCTION** |  |  |  |
| 2 | **Background** | Explain the scientific background and rationale for the reported study. What is the exposure? Is a potential causal relationship between exposure and outcome plausible? Justify why MR is a helpful method to address the study question |  | Utilizing summary-level Quantitative Trait Loci (QTL) data from blood samples at three levels—methylation, RNA, and protein—we employed MR and colocalization analyses to uncover the causal relationships between mitochondrial-related genes and T2DM as well as its complications. This approach adeptly navigates the challenges of reverse causation and confounding factors, which are prevalent in observational studies. |
| 3 | **Objectives** | State specific objectives clearly, including pre-specified causal hypotheses (if any). State that MR is a method that, under specific assumptions, intends to estimate causal effects |  | Mendelian Randomization (MR) leverages genetic variations as instrumental variables (IVs) to deduce the causal relationships between exposures and outcomes. This approach adeptly navigates the challenges of reverse causation and confounding factors, which are prevalent in observational studies. The underlying mechanism is the random allocation of genetic variations at the time of conception, effectively mitigating confounders and reverse causality. |
|  | **METHODS** |  |  |  |
| 4 | **Study design and data sources** | Present key elements of the study design early in the article. Consider including a table listing sources of data for all phases of the study. For each data source contributing to the analysis, describe the following: |  |  |
|  | a) | Setting: Describe the study design and the underlying population, if possible. Describe the setting, locations, and relevant dates, including periods of recruitment, exposure, follow-up, and data collection, when available. |  | Figure 1. |
|  | b) | Participants: Give the eligibility criteria, and the sources and methods of selection of participants. Report the sample size, and whether any power or sample size calculations were carried out prior to the main analysis |  | Supplementary Table 1. |
|  | c) | Describe measurement, quality control and selection of genetic variants |  | We selected top cis-SNP within a 1000 kb range around the gene body or CpG island, achieving the whole genome significance (p < 5.0 × 10-8). SNPs exhibiting differences in allele frequencies greater than 0.2 between any of the datasets, including the linkage disequilibrium reference, QTL, and outcome data, were excluded to ensure data integrity. |
|  | d) | For each exposure, outcome, and other relevant variables, describe methods of assessment and diagnostic criteria for diseases |  | In this study, all T2DM diagnoses were standardized using ICD-9 or ICD-10 criteria. For summary-level GWAS data related to T2DM complications, the datasets were collected from the FinnGen R9 database (https://r9.finngen.fi/) (24), including T2DM with coma (ICD-10: E11.0), ketoacidosis (ICD-10: E11.1), kidney complications (ICD-10: E11.2), retinal complications (ICD-10: E11.3), neuropathy (ICD-10: E11.4), and peripheral circulatory complications (ICD-10: E11.5). |
|  | e) | Provide details of ethics committee approval and participant informed consent, if relevant |  | The data in this study is de-identified and collected from the public databases. The approvement and informed consent can be found in the original article cited in this study. Therefore, no further approvement and consent are needed. |
| 5 | **Assumptions** | Explicitly state the three core IV assumptions for the main analysis (relevance, independence and exclusion restriction) as well assumptions for any additional or sensitivity analysis |  | Mendelian Randomization (MR) leverages genetic variations as instrumental variables (IVs) to deduce the causal relationships between exposures and outcomes. This approach adeptly navigates the challenges of reverse causation and confounding factors, which are prevalent in observational studies. The underlying mechanism is the random allocation of genetic variations at the time of conception, effectively mitigating confounders and reverse causality. |
| 6 | **Statistical methods: main analysis** | Describe statistical methods and statistics used |  |  |
|  | a) | Describe how quantitative variables were handled in the analyses (i.e., scale, units, model) |  | Genetically predicted each standard deviation (SD) increase in gene methylation, the odds ratios (ORs) for causal effects ranged from 0.868 (95% CI = 0.843-0.894) for cg03977443 (ACSL1) to 1.173 (95% CI = 1.135-1.212) for cg24361350 (DNAJC11). |
|  | b) | Describe how genetic variants were handled in the analyses and, if applicable, how their weights were selected |  | We selected top cis-SNP within a 1000 kb range around the gene body or CpG island, achieving the whole genome significance (p < 5.0 × 10-8). SNPs exhibiting differences in allele frequencies greater than 0.2 between any of the datasets, including the linkage disequilibrium reference, QTL, and outcome data, were excluded to ensure data integrity. |
|  | c) | Describe the MR estimator (e.g. two-stage least squares, Wald ratio) and related statistics. Detail the included covariates and, in case of two-sample MR, whether the same covariate set was used for adjustment in the two samples |  | SMR is a sophisticated analytical method used in genetics and epidemiology to investigate potential causal relationships between traits, typically between QTLs and diseases. |
|  | d) | Explain how missing data were addressed |  | We selected top cis-SNP within a 1000 kb range around the gene body or CpG island, achieving the whole genome significance (p < 5.0 × 10-8). SNPs exhibiting differences in allele frequencies greater than 0.2 between any of the datasets, including the linkage disequilibrium reference, QTL, and outcome data, were excluded to ensure data integrity. |
|  | e) | If applicable, indicate how multiple testing was addressed |  | To control for the false discovery rate (FDR) and maintain its threshold at 0.05, we employed the Benjamini-Hochberg procedure for p-value adjustment. |
| 7 | **Assessment of assumptions** | Describe any methods or prior knowledge used to assess the assumptions or justify their validity |  | To evaluate the IVs pleiotropy, we applied the Heterogeneity in Dependent Instrument (HEIDI) test. A p-value of HEIDI test less than 0.05 was indicative of potential pleiotropic effects, leading to the exclusion of the gene from further consideration. |
| 8 | **Sensitivity analyses and additional analyses** | Describe any sensitivity analyses or additional analyses performed (e.g. comparison of effect estimates from different approaches, independent replication, bias analytic techniques, validation of instruments, simulations) |  | A p-value of HEIDI test less than 0.05 was indicative of potential pleiotropic effects, leading to the exclusion of the gene from further consideration. |
| 9 | **Software and pre-registration** |  |  |  |
|  | a) | Name statistical software and package(s), including version and settings used |  | These analyses were conducted using the SMR software (version 1.3.1). All these analyses were conducted using the 'coloc' package (version 5.2.3) in the R software (version 4.1.2). |
|  | b) | State whether the study protocol and details were pre-registered (as well as when and where) |  | The data in this study is de-identified and collected from the public databases. The approvement and informed consent can be found in the original article cited in this study. Therefore, no further approvement and consent are needed. |
|  | **RESULTS** |  |  |  |
| 10 | **Descriptive data** |  |  |  |
|  | a) | Report the numbers of individuals at each stage of included studies and reasons for exclusion. Consider use of a flow diagram |  | NA |
|  | b) | Report summary statistics for phenotypic exposure(s), outcome(s), and other relevant variables (e.g. means, SDs, proportions) |  | Supplementary Table 1 |
|  | c) | If the data sources include meta-analyses of previous studies, provide the assessments of heterogeneity across these studies |  | NA |
|  | d) | For two-sample MR:  i.  Provide justification of the similarity of the genetic variant-exposure associations between the exposure and outcome samples  ii.  Provide information on the number of individuals who overlap between the exposure and outcome studies |  | Supplementary Tables 2-7 |
| 11 | **Main results** |  |  |  |
|  | a) | Report the associations between genetic variant and exposure, and between genetic variant and outcome, preferably on an interpretable scale |  | Supplementary Tables 2-7 |
|  | b) | Report MR estimates of the relationship between exposure and outcome, and the measures of uncertainty from the MR analysis, on an interpretable scale, such as odds ratio or relative risk per SD difference |  | Figure 2-4  Tables 1-3 |
|  | c) | If relevant, consider translating estimates of relative risk into absolute risk for a meaningful time period |  | Figure 2-4  Tables 1-3 |
|  | d) | Consider plots to visualize results (e.g. forest plot, scatterplot of associations between genetic variants and outcome versus between genetic variants and exposure) |  | Figure 2-4 |
| 12 | **Assessment of assumptions** |  |  |  |
|  | a) | Report the assessment of the validity of the assumptions |  | Table 1; Supplementary Tables 2-7 |
|  | b) | Report any additional statistics (e.g., assessments of heterogeneity across genetic variants, such as *I^2^*, Q statistic or E-value) |  | Table 1; Supplementary Tables 2-7 |
| 13 | **Sensitivity analyses and additional analyses** |  |  |  |
|  | a) | Report any sensitivity analyses to assess the robustness of the main results to violations of the assumptions |  | Table 1; Supplementary Tables 2-7 |
|  | b) | Report results from other sensitivity analyses or additional analyses |  | Table 1; Supplementary Tables 2-7 |
|  | c) | Report any assessment of direction of causal relationship (e.g., bidirectional MR) |  | NA |
|  | d) | When relevant, report and compare with estimates from non-MR analyses |  | NA |
|  | e) | Consider additional plots to visualize results (e.g., leave-one-out analyses) |  | NA |
|  | **DISCUSSION** |  |  |  |
| 14 | **Key results** | Summarize key results with reference to study objectives |  | In this study, we employed MR and colocalization analysis to uncover the causal associations between mitochondrial-related genes in blood at the levels of methylation, RNA, and protein with T2DM and its complications. By integrating multi-omics evidence of MR and colocalization results, 18 causal mitochondrial-related genes were identified and validated in specific tissues. We also investigated the underlying regulation network of these genes at multi-omics levels. Furthermore, we delved deeper into the potential functions, their druggability, and associations with other diseases through enrichment analysis, targeted drug searching, and Phe-MR analysis, respectively. |
| 15 | **Limitations** | Discuss limitations of the study, taking into account the validity of the IV assumptions, other sources of potential bias, and imprecision. Discuss both direction and magnitude of any potential bias and any efforts to address them |  | However, recognizing the limitations aids in correctly interpreting the results. Firstly, the analysis was limited to European ancestry, reducing the generalizability of our findings to other populations. Secondly, the limited sample sizes in GWAS cohorts at the levels of methylation, RNA, and protein may introduce potential biases into the results. Thirdly, we utilized the largest GWAS cohort of T2DM in European populations to date. Since it encompasses the majority of European T2DM cohorts in a meta-analysis, there was no suitable dataset for validation. Fourth, the datasets related to T2DM complications were still limited in terms of diseases and sample sizes, suggesting caution in interpreting the causal relationships. Fifth, to mitigate horizontal pleiotropy, cis-SNPs were used as IVs, possibly overlooking the effects of trans-regulation, which might be crucial for some protein-coding genes. Sixth, this study may have ignored some meaningful genes lacking suitable IVs for MR analysis. Last but not least, these causal associations are based solely on MR and colocalization analyses, thus requiring further validation through population-based studies and in vitro/in vivo experiments. |
| 16 | **Interpretation** |  |  |  |
|  | a) | Meaning: Give a cautious overall interpretation of results in the context of their limitations and in comparison with other studies |  | In summary, this study integrated MR and colocalization analyses to identify 18 causal mitochondrial-related genes associated with T2DM across methylation, RNA, and protein levels, and validated them in specific tissues. Among these genes, TUFM, HIBCH, and ISCA2 could serve as biomarkers and potential therapeutic targets for mitochondrial therapy in T2DM. These findings highlight the crucial role of mitochondrial dysfunction in the pathogenesis of T2DM and its complications. |
|  | b) | Mechanism: Discuss underlying biological mechanisms that could drive a potential causal relationship between the investigated exposure and the outcome, and whether the gene-environment equivalence assumption is reasonable. Use causal language carefully, clarifying that IV estimates may provide causal effects only under certain assumptions |  | Utilizing enrichment analysis of KEGG pathways and mitochondrial pathways, the identified mitochondrial-related genes and their potential mechanisms in T2DM were explored. These genes were implicated not only in various nutrient metabolisms, such as carbohydrates, fatty acids, and amino acids, but also in mitochondrial dynamics and surveillance, including mitophagy, autophagy, and apoptosis. Additionally, two diseases closely associated with T2DM, non-alcoholic fatty liver disease and diabetic cardiomyopathy, were also enriched. Given the critical roles of pancreatic β-cell dysfunction and insulin resistance in T2DM, we hypothesize that these genes may contribute to β-cell dysfunction and insulin resistance not only by directly affecting nutrient metabolisms but also through mitophagy, autophagy, and apoptosis (11-13). This further emphasizes the significance of studying mitochondrial dysfunction, particularly mitophagy, autophagy, and apoptosis, in T2DM and its complications. |
|  | c) | Clinical relevance: Discuss whether the results have clinical or public policy relevance, and to what extent they inform effect sizes of possible interventions |  | Through searching the DrugBank and ChEMBL databases, we explored the potential drugs of the 18 causal mitochondrial-related genes previously identified. Among these genes, only 8 were linked to relevant targeted drugs, including TUFM, HIBCH, COMT, GATM, DCXR, HADHA, SLC25A13, and MTHFS (Table S10). It was discovered that zinc acts as a cofactor for TUFM, thereby influencing its biological functions. Quercetin has been identified as targeting HIBCH, though its effects remain unclear. A variety of inhibitors, substrates, and inducers are known to affect the activity of COMT. As a protective factor for T2DM, the inducer Nylidrin for COMT may represent a potential therapeutic option for T2DM. The specific actions for the targeted drugs related to the remaining five druggable genes are still under investigation. To assess the beneficial or detrimental effects of the causal mitochondrial-related genes on other diseases, we employed the Phe-MR analysis to investigate the causal relationships between these genes and 784 disease phenotypes collected from the UK Biobank. A total of 849 gene-disease associations reached marginal significance (p < 0.05) (Table S11). However, only 13 causal associations remained statistically significant after FDR correction (FDR < 0.1) (Table 3). TUFM, a risk factor for T2DM, was positively associated not only with obesity and overweight but also with varicose veins and rheumatoid arthritis, underscoring the importance as a therapeutic target. ACADVL was found to be associated with a reduced risk of essential hypertension, nonspecific chest pain, and skin non-epithelial cancer. Therefore, targeting ACADVL may be beneficial for patients with T2DM who also suffer from hypertension. Targeting SLC25A13, while potentially lowering the risk of T2DM, may lead to neutropenia and a range of musculoskeletal diseases, such as arthropathy, intervertebral disc degeneration, and joints disorders. |
| 17 | **Generalizability** | Discuss the generalizability of the study results (a) to other populations, (b) across other exposure periods/timings, and (c) across other levels of exposure |  | Firstly, the analysis was limited to European ancestry, reducing the generalizability of our findings to other populations. |
|  | **OTHER INFORMATION** |  |  |  |
| 18 | **Funding** | Describe sources of funding and the role of funders in the present study and, if applicable, sources of funding for the databases and original study or studies on which the present study is based |  | This research received no external funding. |
| 19 | **Data and data sharing** | Provide the data used to perform all analyses or report where and how the data can be accessed, and reference these sources in the article. Provide the statistical code needed to reproduce the results in the article, or report whether the code is publicly accessible and if so, where |  | Supplementary Table 1 |
| 20 | **Conflicts of Interest** | All authors should declare all potential conflicts of interest |  | The authors declare that they have no competing interests. |

This checklist is copyrighted by the Equator Network under the Creative Commons Attribution 3.0 Unported (CC BY 3.0) license.

1. Skrivankova VW, Richmond RC, Woolf BAR, Yarmolinsky J, Davies NM, Swanson SA, et al. Strengthening the Reporting of Observational Studies in Epidemiology using Mendelian Randomization (STROBE-MR) Statement. JAMA. 2021;under review.

2. Skrivankova VW, Richmond RC, Woolf BAR, Davies NM, Swanson SA, VanderWeele TJ, et al. Strengthening the Reporting of Observational Studies in Epidemiology using Mendelian Randomisation (STROBE-MR): Explanation and Elaboration. BMJ. 2021;375:n2233.
